# The impact of early adversity on the cerebral cortex - a Mendelian randomization study

**DOI:** 10.3389/fnins.2023.1283159

**Published:** 2023-10-27

**Authors:** Zhen Wang, Jing Zou, Le Zhang, Jinghua Ning, Xin Zhang, Bei Jiang, Yi Liang, Yuzhe Zhang

**Affiliations:** ^1^College of Basic Medical Sciences, Dali University, Dali, Yunnan, China; ^2^The First Affiliated Hospital of Dali University, Dali, Yunnan, China; ^3^Yunnan Key Laboratory of Screening and Research on Anti-pathogenic Plant Resources from West Yunnan (Cultivation), Dali, Yunnan, China; ^4^Princess Margaret Cancer Centre, University Health Network, TMDT-MaRS Centre, Toronto, ON, Canada

**Keywords:** early adversity, cerebral cortex, Mendelian randomization (MR), lateral occipital cortex, brain structure

## Abstract

**Background:**

The early adversity is associated with a series of negative outcomes in adulthood, and the impact on the cerebral cortex may be one of the fundamental causes of these adverse consequences in adulthood. In this study, we aim to investigate the causal relationship between early adversity and changes in cerebral cortex structure using Mendelian randomization (MR) analysis.

**Methods:**

The GWAS summary statistics of 6 early adversity traits were obtained from individuals of European ancestry in the UK Biobank. The GWAS summary statistics of 34 known functional cortical regions were obtained from the ENIGMA Consortium. Causal relationships between the adversity factors and brain cortical structure were assessed using the inverse-variance weighted (IVW), MR-Egger, and weighted median methods, with IVW being the primary evaluation method. Cochran’s *Q*-test, MR-PRESSO, leave-one-out analysis, and funnel plot examination were employed to detect potential heterogeneity and pleiotropy, as well as to identify and exclude outliers.

**Results:**

At a global level, no causal relationship was found between early adversity and cortical thickness (TH) or surface area (SA) of the brain. However, at the regional level, early adversity was found to potentially influence the TH of the caudal anterior cingulate, superior temporal, entorhinal, paracentral, lateral occipital, banks of the superior temporal sulcus, and supramarginal regions, as well as the SA of the pars triangularis, lateral occipital, parahippocampal, medial orbitofrontal, and isthmus cingulate regions. All findings were nominally significant and passed sensitivity analyses, with no significant heterogeneity or pleiotropy detected.

**Discussion:**

Our study provides evidence for the association between early adversity and alterations in brain cortical structure, which may serve as a foundation for certain mental disorders. Furthermore, magnetic resonance imaging (MRI) might be considered as a promising tool to aid healthcare professionals in identifying individuals with a history of adverse experiences, allowing for early interventions.

## Introduction

The increasing prevalence of adverse childhood experiences (ACEs) has emerged as an undeniable public health concern (Hughes et al., 2017; Thompson et al., 2020). According to a report from the U.S. Children’s Bureau, in 2018 alone, approximately 678,000 children experienced abuse and neglect. Among them, 60.8% experienced neglect, 10.7% suffered physical abuse, 7.0% were subjected to sexual abuse, and 15.5% endured two or more forms of maltreatment (Lopez et al., 2021). Studies covering the entire age spectrum of adverse experiences have found that 67–98% of individuals recall at least one adverse childhood experience (Mersky et al., 2013; Campbell et al., 2016). A retrospective study based on the World Mental Health Surveys, involving 24,000 adults, revealed that ACEs doubled the risk of adult-onset mental illness, accounting for 31% of global mental illness cases (McGrath et al., 2017). Additionally, ACEs have also been linked to suicide, substance abuse (Friestad et al., 2014), risky sexual behaviors (VanderEnde et al., 2018), chronic non-communicable diseases, domestic violence, and poorer overall physical and mental health outcomes (Brown et al., 2009; Duke et al., 2010; Mersky et al., 2013; Espeleta et al., 2018).

The cerebral cortex serves as the foundation for human complex cognitive abilities. Variations in cortical surface area (SA) and thickness (TH) are associated with neural, psychological, and behavioral characteristics (Grasby et al., 2020). Numerous research studies have reported an association between childhood maltreatment and changes in brain structure and function. In both laboratory and population-based studies, specific regions such as the adult hippocampus or the anterior cingulate cortex, as well as pathways like the corpus callosum, have been consistently implicated in relation to childhood maltreatment (Teicher et al., 2016). Mechanistically, some researchers propose that experiences occurring during prenatal development and the early years of life, when the developing brain is highly susceptible to environmental influences, may lead to permanent changes in brain structure and function through epigenetic modifications (e.g., alterations in DNA structure and chromatin function) (Miguel et al., 2019). However, the causal relationship between early adversity and the cerebral cortex is still not fully understood.

Magnetic Resonance Imaging (MRI) is a sensitive tool that enables the study of changes in brain structure. However, conducting experiments involving early adversity, such as randomly selecting pregnant women to smoke or randomly subjecting children to abuse, would be highly unethical. Moreover, traditional experimental methods require substantial costs, have long durations, and often struggle to eliminate confounding factors and the influence of reverse causality. In this study, we employ Mendelian randomization (MR) as a method to explore the potential causal relationship between early adversity and changes in cerebral cortex. MR utilizes genetic variants as instrumental variables (IVs) to investigate causal relationships between risk factors and outcomes, thereby overcoming confounding factors and reverse causality inherent in observational studies (Davey Smith and Hemani, 2014). Importantly, this approach is ethically permissible. Specifically, we will use MR to investigate the causal relationship between early adversity risk factors (including “Physically abused by family as a child,” “Sexually molested as a child,” “Adopted as a child,” “Felt hated by family member as a child,” “Maternal smoking around birth,” “Part of a multiple birth”) and, the SA and TH of both the global cerebral cortex and 34 known functional regions.

## Materials and methods

### Data on early adversity

The GWAS summary statistics for early adversity were based on European ancestry persons aged 45–82 and 57% were women from the UK Biobank (Bycroft et al., 2018). Data on selected features such as “Maternal smoking around birth” (*N* = 309,942, phenotype code: 1787), “Part of a multiple birth” (*N* = 355,467, phenotype code: 1777), and “Adopted as a child” (*N* = 360,450, phenotype code: 1767) were obtained from a touchscreen questionnaire, which involved approximately 500,000 participants, using the ACEs system (Davis et al., 2020). The relevant information can be found in the” Early life and reproductive factor” category of the UK Biobank’s website.^1^ Data regarding “Sexually molested as a child” (*N* = 116,773, phenotype code: 20490), “Felt hated by family member as a child” (*N* = 117,749, phenotype code: 20487) and “Physically abused by family as a child” (*N* = 117,838, phenotype code: 20488) are derived from the UK Biobank on-line “Thoughts and Feelings” mental health questionnaire, which covered 157,300 participants (Gheorghe et al., 2021). Detailed information regarding traumatic events can be found under the “Traumatic events” category on the UK Biobank’s website.^2^ The summary information on the sources of early adversity can be referred to in Supplementary Table S1. All participants of the UK Biobank have provided written informed consent, and the study has received approval from the local research ethics committee.

### Data on cerebral cortex

The GWAS summary statistics of cerebral cortex regions were derived from a diverse sample of 51,665 individuals recruited from 60 cohorts worldwide (Supplementary Table S2), with a predominant European descent (~94%) (Grasby et al., 2020). The Cortical SA and TH measures were obtained from structural brain MRI scans of 34 regions with known functional specializations, as defined by the Desikan-Killiany atlas. The regional boundaries were delineated based on gyral anatomy labeled between the depths of the sulci (Desikan et al., 2006). To account for individual variations in overall brain size, the regional SA and TH measures were adjusted using their respective global measures. In our study, both the cortical TH and SA of the global levels of cortical region and 34 brain cortical regions with established functional specializations were analyzed, with or without the inclusion of global weighted data. The GWAS data pertaining to all brain structures were obtained from the ENIGMA Consortium.^3^ Participants in all cohorts gave written informed consent, and each site obtained approval from local research ethics committees or institutional review boards.

### Instrumental variable

The selection of IVs should be based on certain criteria to ensure a strong correlation with the exposure and minimize bias in the results. Therefore, the following criteria were followed for IV selection: (1) Single nucleotide polymorphisms (SNPs) with a significance threshold (*p* < 5.0 × 10^−6^) were considered as potential IVs; (2) SNPs with a linkage disequilibrium (LD) measure, *r*^2^ < 0.001 (clumping distance = 10,000 kb), were retained; (3) IVs must exhibit a strong correlation with the exposure, indicated by an F statistic >10. The F statistic can be calculated using the formula F = R2(N-2)/(1-R2), where R2 represents the variance in early adversity explained by the genetic instrument, and *N* represents the sample size (Burgess and Thompson, 2011). To calculate R2, the following formula was used: (2 × eaf × (1-eaf) × beta^2^)/[(2 × eaf × (1-eaf) × beta^2^) + (2 × eaf × (1-eaf) × *N* × se^2^)], where eaf is the effect allele frequency, beta is the estimated genetic effect, *N* is the sample size of the GWAS, and se is the standard error of the genetic effect (Papadimitriou et al., 2020).

### Statistical analysis

The study employs a two-sample MR analysis. MR is an epidemiological method that utilizes genetic variation as IVs to address potential confounding factors and reverse causality in the study design. An effective MR analysis relies on three main assumptions: (1) The IVs must be strongly associated with the exposure factor; (2) The IVs should be unrelated to any potential confounding factors; (3) There should be no correlation between the IVs and the outcome.

The inverse variance weighting (IVW) method, which is a standard approach in MR analysis, was utilized as the primary analytical method in this study to investigate the relationship between early adversity and cortical brain structure. The IVW method does not require individual-level data and can directly estimate the causal effect using summary statistics. If the genetic instruments are uncorrelated, the estimated effect size using IVW will be equivalent to that obtained from the two-stage least squares (2SLS) method using individual-level data (Burgess et al., 2016). However, like other MR methods, the IVW method is also susceptible to weak instrument bias. To address this issue, two additional MR methods were employed in this study: MR-Egger, a weighted regression method that introduces an intercept to accommodate horizontal pleiotropy; and weighted median, a method that permits the utilization of potentially invalid instruments under the assumption that at least half of the instruments employed in the MR analysis are valid (Bowden et al., 2015, 2016).

The statistical heterogeneity of SNPs was examined using the Cochran’s *Q*-test, with a significance level of *p* < 0.05 indicating significant heterogeneity. Several sensitivity analyses were conducted to investigate and correct for potential horizontal pleiotropy in causal estimates. The MR-Egger intercept test was also utilized to assess the potential presence of horizontal pleiotropy. MR-PRESSO was employed to evaluate the presence of pleiotropy, and if detected, outlying SNPs were excluded and the estimates were re-evaluated (Verbanck et al., 2018). Furthermore, to ensure the stability of the MR analysis results and mitigate the influence of individual SNPs on the overall findings, leave-one-out analysis was also conducted. In addition, a funnel plot was employed with the aim of assessing the presence of directional pleiotropy. A symmetrical funnel plot suggests the absence of potential directional pleiotropy.

To assess whether the IVs were associated with other potential confounding factors (*r*^2^ > 0.8, *p*-value <5 × 10^−8^), we also employed the PhenoScanner^4^ web tool. The tool was used to evaluate potential associations between IVs and a range of potential confounders, including mental disorders (Gaser et al., 2004), smoking (Corley et al., 2019), alcohol consumption (Mavromatis et al., 2022), hypertension (Newby et al., 2022), intelligence (Deary et al., 2022), and educational attainment (Cox et al., 2016). Any SNPs that showed significant associations with these factors and could potentially introduce confounding were removed from the IVs (Staley et al., 2016).

### Statistics

All analyses were performed using the TwoSampleMR package (version 0.5.6) in R language (version 4.1.2). In the global level MR analysis, a significant *p*-value was defined as less than 0.05. To correct for multiple comparisons since a total of 816 regional level MR analyses were conducted, the Bonferroni adjustment was applied (*p*-value threshold: 0.05/816 outcomes = 6.13 × 10^−5^) (Glickman et al., 2014). Additionally, a *p*-value less than 0.05 was considered nominally significant.

## Results

**Figure 1:**
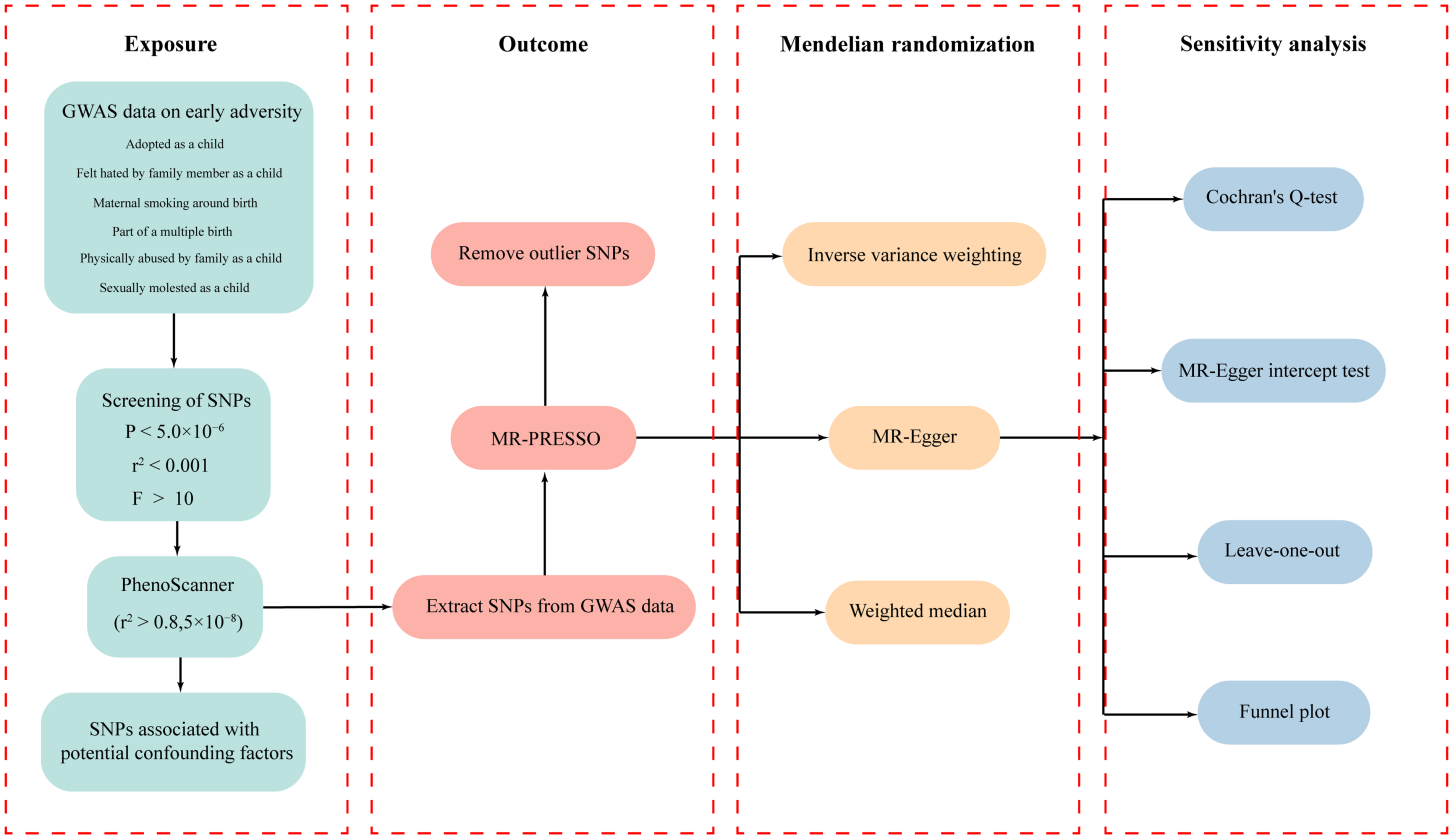
The workflow diagram of this study.

The workflow diagram of this study is illustrated in Figure 1. All IVs related to early adversity were screened based on a threshold of *p* < 5.0 × 10^−6^ and (LD) *r*^2^ < 0.001. The specific information regarding all IVs related to early adversity can be found in Supplementary Tables S3-S8. According to the evaluation results from PhenoScanner, some SNPs that may be associated with confounding factors were removed. The F statistics for all SNPs were greater than 10, indicating strong correlation between the IVs and the exposure factors.

The MR analysis exploring the association between early adversity-related exposures and global levels of cortical SA and TH showed no significant correlation between early adversity exposure and changes in global SA and TH (Supplementary Table S9).

**Figure 2:**
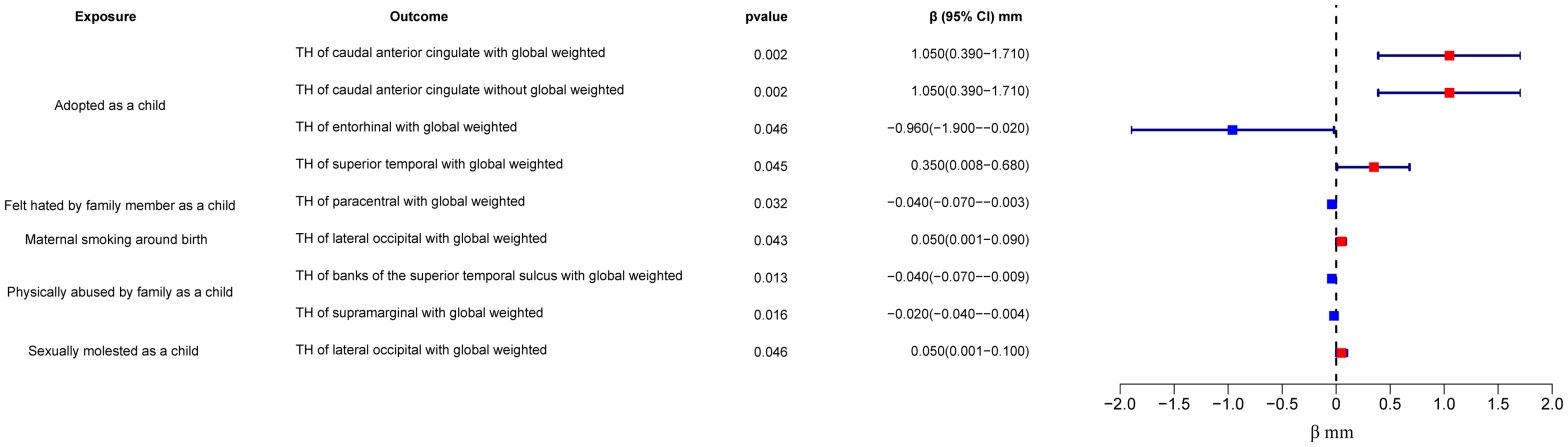
Forest plot of the IVW analysis results for early life adversity and cortical TH. TH, thickness.

**Figure 3:**
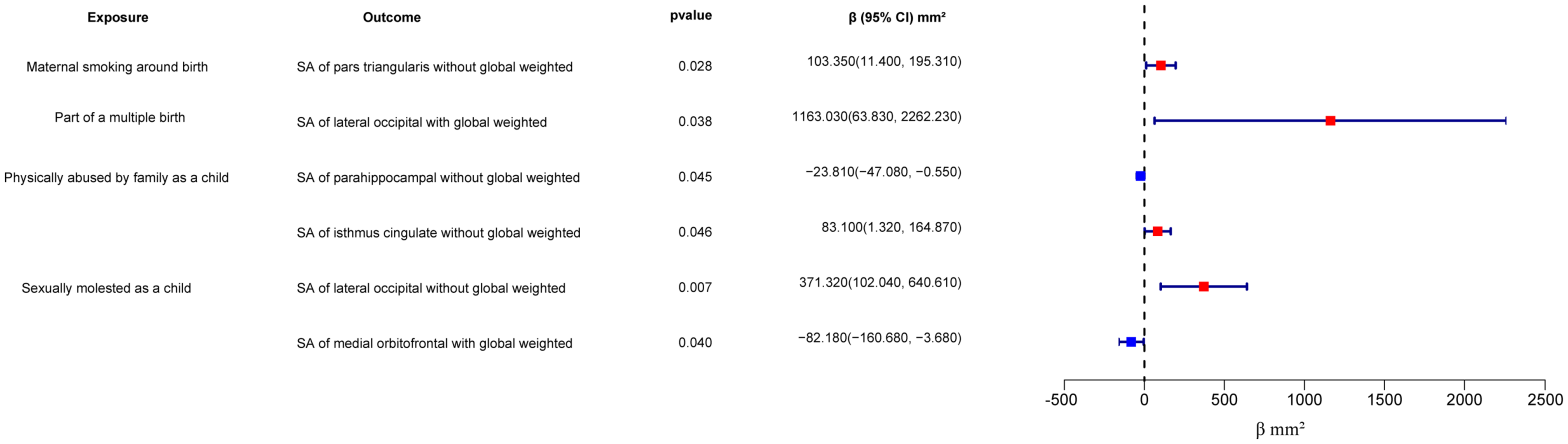
Forest plot of the IVW analysis results for early life adversity and cortical SA. SA, surface area.

**Figure 4:**
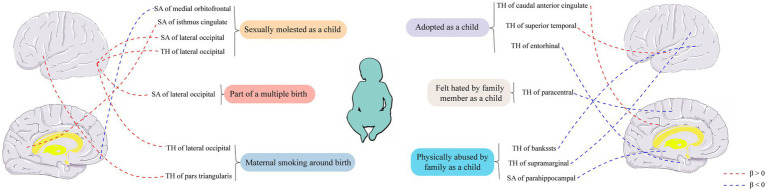
Causal relationships between six early-life adversity exposure factors and cortical structure were analyzed using Mendelian randomization. SA, surface area; TH, thickness.

In the regional-level MR analysis, we observed associations between the factor “Adopted as a child” and the TH of the caudal anterior cingulate with global weighted (*β* = 1.05 mm, 95% CI: 0.39 mm to 1.71 mm, *p* = 0.002, unless otherwise specified, default displaying IVW results), the TH of the caudal anterior cingulate without global weighted (*β* = 1.05 mm, 95% CI: 0.39 mm to 1.71 mm, *p* = 0.002), the TH of the superior temporal with global weighted (*β* = 0.35 mm, 95% CI: 0.008 mm to 0.68 mm, *p* = 0.045), and the TH of the entorhinal with global weighted (*β* = −0.96 mm, 95% CI: −1.9 mm to −0.02 mm, *p* = 0.046) (Figures 2–4; Table 1, Supplementary Figure S1). It is worth noting that the results of TH of the caudal anterior cingulate with global weighted and the TH of the caudal anterior cingulate without global weighted analyses are identical, suggesting that the inclusion of global weighted did not affect the SNPs used in our study. The associations between “Adopted as a child” and the TH of the caudal anterior cingulate with global weighted (*β* = 0.91 mm, 95% CI: 0.05 mm to 1.78 mm, *p* = 0.037), the TH of the caudal anterior cingulate without global weighted (*β* = 0.91 mm, 95% CI: 0.05 mm to 1.78 mm, *p* = 0.037), and the TH of the entorhinal with global weighted (*β* = −1.32 mm, 95% CI: −2.62 mm to −0.02 mm, *p* = 0.047) were also supported by the weighted median method (Supplementary Table S10). Furthermore, the directions of effect (*β* values) were consistent across all three MR methods. For all the nominally significant results, Cochran’s *Q*-test did not detect any heterogeneity (Supplementary Table S12). Moreover, the *p*-values of the MR-Egger intercept exceeded 0.05, which implies the absence of intercept values (Supplementary Table S11). Additionally, no outliers were found in this study according to the leave-one-out sensitivity test and funnel plots (Supplementary Figure S2-S4).

**Table 1:** Nominal positive results between early adversity and the cerebral cortex in Mendelian randomization.

Exposure	Outcome	nSNP	β (95% IC) (mm/mm^2^)	IVW-derived *p* value	Cochran’s Q-derived *p* value	MR-Egger intercept- derived *p* value
Adopted as a child	TH of caudal anterior cingulate with global weighted	13	1.05 (0.39 to 1.71)	0.002	0.96	0.77
	TH of caudal anterior cingulate without global weighted	13	1.05 (0.39 to 1.71)	0.002	0.96	0.77
	TH of superior temporal with global weighted	12	0.35 (0.008 to 0.68)	0.045	0.96	0.33
	TH of entorhinal with global weighted	14	−0.96 (−1.9 to −0.02)	0.046	0.63	0.3
Felt hated by family member as a child	TH of paracentral with global weighted	11	−0.04 (−0.07 to −0.003)	0.032	0.53	0.77
Maternal smoking around birth	SA of pars triangularis without global weighted	30	103.35 (11.4 to 195.31)	0.028	0.89	0.52
	TH of lateral occipital with global weighted	31	0.05 (0.001 to 0.09)	0.043	0.94	0.32
Part of a multiple birth	SA of lateral occipital with global weighted	9	1163.03 (63.83 to 2262.23)	0.038	0.46	0.41
Physically abused by family as a child	TH of banks of the superior temporal sulcus with global weighted	21	−0.04 (−0.07 to −0.009)	0.013	0.94	0.74
	TH of supramarginal with global weighted	23	−0.02 (−0.04 to −0.004)	0.016	0.67	0.51
	SA of parahippocampal without global weighted	22	−23.81 (−47.08 to −0.55)	0.045	0.58	0.52
Sexually molested as a child	SA of lateral occipital without global weighted	9	371.32 (102.04 to 640.61)	0.007	0.76	0.93
	SA of medial orbitofrontal with global weighted	7	−82.18 (−160.68 to −3.68)	0.040	0.98	0.91
	SA of isthmus cingulate without global weighted	8	83.1 (1.32 to 164.87)	0.046	0.85	1
	TH of lateral occipital with global weighted	7	0.05 (0.0008 to 0.1)	0.046	0.99	0.79

IVW, Inverse-variance weighted; IC, Confidence interval; SA, surface area; TH, thickness.

The factor “Felt hated by family member as a child” might be associated with a reduction in the TH of the paracentral with global weighted (*β* = −0.04 mm, 95% CI: −0.07 mm to −0.003 mm, *p* = 0.032), and this result is supported by the Weighted median method as well (*β* = −0.05 mm, 95% CI: −0.10 mm to −0.004 mm, *p* = 0.032).

On the other hand, “Maternal smoking around birth” was found to potentially affect the SA of the pars triangularis without global weighted (*β* = 103.35 mm^2^, 95% CI: 11.4 mm^2^ to 195.31 mm^2^, *p* = 0.028), as well as the TH of the lateral occipital region with global weighted (*β* = 0.05 mm, 95% CI: 0.001 mm to 0.09 mm, *p* = 0.043). The weighted median method also supported these findings obtained from the IVW method. However, the MR-Egger method yielded a substantial deviation from the IVW results, indicating an opposite direction of the *β* value.

Compared to singletons, the “Part of a multiple birth” factor potentially increased the SA of the lateral occipital with global weighted (*β* = 1163.03 mm^2^, 95% CI: 63.83 mm^2^ to 2262.23 mm^2^, *p* = 0.038). Similar results were also observed with the Weighted median method (*β* = 1456.84 mm^2^, 95% CI: 79.24 mm^2^ to 2834.44 mm^2^, *p* = 0.038), and all the results had consistent *β* directions.

“Physically abused by family as a child” might be associated with a decreased cortical TH and SA, including the TH of the banks of the superior temporal sulcus with global weighted (*β* = −0.04 mm, 95% CI: −0.07 mm to −0.009 mm, *p* = 0.013), the TH of the supramarginal with global weighted (*β* = −0.02 mm, 95% CI: −0.04 mm to −0.004 mm, *p* = 0.016), and the SA of the parahippocampal without global weighted (*β* = −23.81 mm^2^, 95% CI: −47.08 mm^2^ to −0.55 mm^2^, *p* = 0.045). Results from weighted median also supported the possible association between childhood abuse and lower TH of the banks of the superior temporal sulcus with global weighted (*β* = −0.05 mm, 95% CI: −0.09 mm to −0.002 mm, *p* = 0.041) and the TH of the supramarginal with global weighted (*β* = −0.03 mm, 95% CI: −0.06 mm to −0.008 mm, *p* = 0.012).

“Sexually molested as a child” may lead to an increase in the SA of the lateral occipital without global weighted (*β* = 371.32 mm^2^, 95% CI: 102.04 mm^2^ to 640.61 mm^2^, *p* = 0.007) and the TH of the lateral occipital with global weighted (*β* = 0.05 mm, 95% CI: 0.0008 mm to 0.1 mm, *p* = 0.046), as well as affect the SA of the medial orbitofrontal with global weighted (*β* = −82.18 mm^2^, 95% CI: −160.68 mm^2^ to −3.68 mm^2^, *p* = 0.040) and the SA of isthmus cingulate without global weighted (*β* = 83.10 mm^2^, 95% CI: 1.32 mm^2^ to 164.87 mm^2^, *p* = 0.046). Only the result for the SA of the lateral occipital without global weighted (*β* = 432.70 mm^2^, 95% CI: 84.81 mm^2^ to 780.59 mm^2^, *p* = 0.015) obtained support from the Weighted median method.

## Discussion

To the best of our knowledge, this is the first large-scale analysis applying the MR method to examine the causal relationship between early adversity and cerebral cortex. We systematically analyzed the relationships between six early adversity factors and the cerebral cortex using GWAS summary statistics obtained from a population of individuals of European descent gathered by the UK Biobank and ENIGMA Consortium. Although our analysis did not yield significant positive results, it is important to exercise caution when interpreting nominally significant findings.

Conservatively speaking, we found suggestive evidence that early adversity factors may influence the TH or SA of the caudal anterior cingulate, superior temporal, entorhinal, paracentral, lateral occipital, banks of the superior temporal sulcus, supramarginal pars triangularis, lateral occipital, parahippocampal, medial orbitofrontal, and isthmus cingulate regions in the cortical brain. The emergence of certain results has cast doubt on the causal relationship between early adversity and changes in the structure of the cerebral cortex. For instance, although they are nominally positive, experiences of childhood sexual abuse have been associated with increased TH and SA of the lateral occipital cortex. Additionally, maternal smoking during pregnancy and participation in multiple pregnancies have also been linked to increased TH or SA of the lateral occipital cortex. These findings concerning the lateral occipital cortex account for more than a quarter of all positive results. These findings concerning the lateral occipital cortex account for more than a quarter of all the positive results. Instead of considering these results as mere coincidences, we are inclined to believe that there is indeed some causal relationship between early adversity and changes in the cerebral cortex, particularly with the high suspicion that early adversity may impact the lateral occipital cortex. This raises the question of why early adversity would lead to alterations in the structure of the cerebral cortex.

The development of the brain is regulated by genetic factors, but it is also shaped by past experiences, especially during early developmental and sensitive periods (Teicher et al., 2016). One key question is why the brain undergoes shaping, whether it is due to pathological damage or for the purpose of adaptation to the environment. Some examples may provide insights. In a study, it was found that adults who experienced adverse events during childhood had an increased risk of obesity, and these abusive events always preceded the development of obesity. More specifically, the issue itself is not obesity, but rather obesity serves as a self-protective solution to the previous abuse (Felitti et al., 1998; Brown et al., 2019). Similarly, the changes in the cerebral cortex may be viewed as the brain’s adaptive response to adversity, similar to how obesity can be seen as a coping mechanism. Another study indicated that repeated sexual abuse by non-parental adults in women was associated with significant bilateral gray matter volume reduction in the primary visual cortex and visual association cortex (Tomoda et al., 2009). Another report also indicated that childhood sexual abuse was associated with partial thinning of the somatosensory cortex, particularly in regions representing the clitoris and genitals, compared to women who had not experienced sexual abuse before adolescence (Heim et al., 2013). These changes can be understood as adaptive modifications to certain sensory systems and conscious pathways in order to mitigate the impact of external stimuli on the organism, serving as a self-protective mechanism. In fact, the occurrence of childhood abuse is quite common, and the structural changes in the brain can be seen as facilitating the survival and reproduction of individuals in a world that appears to be filled with pain and threat to some extent. Therefore, the changes in brain structure may be a compromise made to adapt to adverse environments.

For most individuals, the experience of being adopted is an emotional trauma. Compared to non-adopted peers, adopted individuals have a higher proportion of psychological issues and show higher levels of adaptation problems within the psychological well-being context (Brodzinsky et al., 2022). In our study, we found that the experience of being adopted in childhood may impact the TH of the caudal anterior cingulate cortex, superior temporal cortex, and entorhinal cortex. The caudal anterior cingulate cortex and superior temporal cortex are, respectively, central to emotions and language (Yi et al., 2019; Mertse et al., 2022), and the experience of being adopted in childhood may lead to increased TH in these regions. This may assist adoptees in better navigating the challenges associated with adoption, as adoption may bring about more emotional distress and communication difficulties compared to biological kin. From a neuroanatomical perspective, the entorhinal cortex has long been recognized as a major relay station providing incoming inputs to the hippocampus (Jones, 1993). The entorhinal-hippocampal system is a primary center for memory in the brain (Mankin and Fried, 2020), and the entorhinal cortex is typically one of the earliest brain regions to exhibit histological changes in Alzheimer’s disease (Igarashi, 2023). In contrast to the thickening observed in the emotional and language centers resulting from adoption experiences, the entorhinal cortex may become thinner, possibly as a protective mechanism of the brain to mitigate the distress associated with negative memories.

Nearly every domain of an individual’s development, such as cognition, language, socio-emotional functioning, and neurobiological development, is affected by experiences of abuse (Cicchetti, 2016). In our study, childhood experiences of physical abuse may result in reduced TH in the banks of the superior temporal sulcus and supramarginal regions, as well as decreased SA in the parahippocampal region. The banks of the superior temporal sulcus are enriched with cortical neurons that are selective for faces, facial parts, and specific facial expressions. Damage to this region can lead to prosopagnosia, a condition characterized by an inability to recognize faces or differentiate between faces (Heywood and Cowey, 1992). The supramarginal and parahippocampal regions, on the other hand, have been implicated in emotion recognition abilities (Wada et al., 2021) and visual spatial processing abilities (Aminoff et al., 2013), respectively. Interestingly, a study on a population from northern Finland also demonstrated a link between early adversity and decreased ability to recognize fearful facial expressions (Lieslehto et al., 2017). Based on our study, it is possible that the decrease in the TH of the banks of the superior temporal sulcus is associated with the reduction of these abilities in individuals who experienced early adversity. The shrinking of these regions may indicate a weakening of these abilities in response to childhood experiences of physical abuse, aiming to mitigate the physical and psychological pain associated with abuse. Childhood experiences of sexual abuse may potentially impact the SA and TH of the lateral occipital, medial orbitofrontal, and isthmus cingulate regions. It is noteworthy that while there were no significant positive findings, the association between childhood sexual abuse and the lateral occipital cortex remains speculative. The lateral occipital cortex is not only involved in visual processing but also plays a role in spatial and shape perception of visual stimuli, ultimately contributing to object recognition and identification processes (Ptak et al., 2014; Lingnau and Downing, 2015). Indeed, childhood sexual abuse tends to be associated with increased TH and SA of the lateral occipital cortex, and other early adverse experiences may also influence the lateral occipital region. For instance, maternal smoking during pregnancy has been linked to increased lateral occipital TH in offspring, and multiple gestations have been associated with enlarged lateral occipital SA. These findings strongly suggest a close relationship between the lateral occipital cortex and early adversity. However, the underlying mechanisms warrant further exploration.

Adverse factors experienced by fetuses in the mother’s body can also lead to various adverse outcomes in adulthood (O'Donnell and Meaney, 2017). Among these factors, maternal smoking during pregnancy is one of the most prevalent and has been linked to an increased risk of cardiovascular diseases and hypertension in offspring (Högberg et al., 2012; Mamun et al., 2012; de Jonge et al., 2013; Perak et al., 2021). Research has shown that maternal smoking during pregnancy can also affect the brain structure of offspring. In a study evaluating brain MRI in children with an average age of 10.1 years, prenatal exposure to smoking was associated with reduced gray matter, white matter volume, and smaller SA compared to non-smoking exposure (Zou et al., 2022). The underlying mechanisms suggest that tobacco toxins can cross the placental barrier, inhibiting the expression of specific brain regulatory genes involved in brain growth, myelination, and neuronal migration, thereby affecting brain structure and function (Salihu et al., 2017). In our study, we found that maternal smoking during pregnancy may lead to an increase in the SA of the pars triangularis and an increase in the TH of the lateral occipital cortex in offspring. This seems to contradict the findings of the aforementioned study, which reported reduced gray matter, white matter volume, and smaller SA associated with prenatal smoking exposure. However, it is important to note that the previous study focused on brain MRI evaluation in children with an average age of 10.1 years (9 to 11 years old), while our study analyzed the human cerebral cortex across all age groups (3.3 to 90.0 years old). Therefore, our results are not in conflict with the findings of that study. The specific mechanisms through which maternal smoking during pregnancy can influence the pars triangularis and lateral occipital cortex across all age groups require further investigation. Multiple gestation, on the other hand, serves as a natural early adversity factor that can restrict fetal growth and increase the risks of premature birth and perinatal complications (Townsend and Khalil, 2018). Mechanistically, compared to singleton pregnancies, the presence of multiple fetuses in the maternal body leads to competition for space and nutrients, which can result in inadequate fetal nutrition and oxygen supply, thereby leading to adverse outcomes. In our study, multiple gestation was associated with a significant increase in the SA of the lateral occipital cortex. However, the specific mechanisms underlying this relationship necessitate further research.

Even though society has begun to allocate more attention to ACEs, the actual prevalence of ACEs remains underestimated, particularly among young children who, due to their limited motor and language skills, often cannot report these adverse experiences (Felitti et al., 1998). In addition, retrospective surveys of ACEs in adults have revealed a higher occurrence of adverse events compared to studies conducted with child populations. This suggests that a significant number of ACEs go unreported. ACEs pose a challenge as they often originate from parents or close relatives and are limited by children’s understanding and language abilities (Thompson et al., 2020). Structural brain MRI scans provide an effective screening method. Given that ACEs can lead to changes in specific cerebral cortex, MRI can assist clinicians in identifying children who have experienced early adversity. Physical activity may serve as a cost-effective behavioral intervention to mitigate the long-term impact of ACEs on brain health. Research indicates that physical activity can influence brain health and intervene in brain structure through various mechanisms, including neurotrophic factors, hypothalamic–pituitary–adrenal axis regulation, inflammatory processes, and epigenetic mechanisms (Donofry et al., 2021). Therefore, early identification of individuals exposed to ACEs can enable clinicians to intervene through physical activity at an earlier stage, potentially mitigating the adverse effects of ACEs.

Our study has several limitations. Firstly, since our MRI data is derived from a predominantly European population spanning all age groups (3.3 to 90.0 years old), our findings are specific to cortical structural changes in this population and may not generalize well to other populations or age groups. Therefore, further analysis using data from different populations or age ranges would enhance the generalizability of the study. Secondly, as our data is sourced from aggregated GWAS data in public databases, there is an inherent difficulty in avoiding sample overlap. However, we calculated that the overlap rate was less than 6.4%, which is acceptable given our sample size. Lastly, our study can only demonstrate an association between early adversity and cortical structural changes, and while we have provided potential explanations for some of the observed changes in cortical structures in the discussion section, the underlying mechanisms still require further investigation.

## Conclusion

In essence, this study constitutes the foremost comprehensive MR exploration into the nexus linking early adversity and cortical SA and TH. Although no statistically significant affirmative results were yielded to establish a significant correlation between early adversity and cortical SA and TH, some nominally positive findings are of particular interest. Specifically, the linkage between the lateral occipital cortex and diverse early adversity factors, with increased SA and TH in this region, warrants further attention. However, it is essential to exercise caution when interpreting other nominally positive results. Our inquiry furnishes evidence supporting the enduring impact of early adversity, comprising traumatic encounters during childhood and *in utero*, on permanent alterations in cortices, which may underpin particular psychiatric conditions.

## Data availability statement

Publicly available datasets were analyzed in this study. This data can be found here: all data used in this work can be acquired from the UK Biobank (http://www.nealelab.is/uk-biobank) and ENIGMA Consortium (https://enigma.ini.usc.edu/).

## Author contributions

ZW: Formal analysis, Investigation, Resources, Visualization, Writing – original draft. JZ: Visualization, Writing – review & editing. LZ: Writing – review & editing. JN: Writing – review & editing. XZ: Writing – review & editing. BJ: Resources, Writing – review & editing. YL: Methodology, Project administration, Writing – review & editing. YZ: Funding acquisition, Methodology, Project administration, Writing – review & editing.

## Footnotes

^1^https://biobank.ctsu.ox.ac.uk/crystal/field.cgi?id=1787

^2^https://biobank.ctsu.ox.ac.uk/crystal/label.cgi?id=145

^3^https://enigma.ini.usc.edu/

^4^http://www.phenoscanner.medschl.cam.ac.uk/
