# Supplementary material for: The impact of early adversity on the cerebral cortex - a Mendelian randomization study
Source: Front Neurosci. 2023 Oct 27;17:1283159. doi: 10.3389/fnins.2023.1283159 (PMC10641447; doi:10.3389/fnins.2023.1283159)
Supplement: Supplementary file 1 [file Data_Sheet_1.docx]

Supplementary Information

**The Impact of Early Adversity on the Cerebral cortex - A Mendelian Randomization Study**

Zhen Wang^1^, Jing Zou^2^, Le Zhang^1^, Jinghua Ning^1^, Xin Zhang^1^ Bei Jiang^3^, Yi Liang^4^* and Yuzhe Zhang^1^*


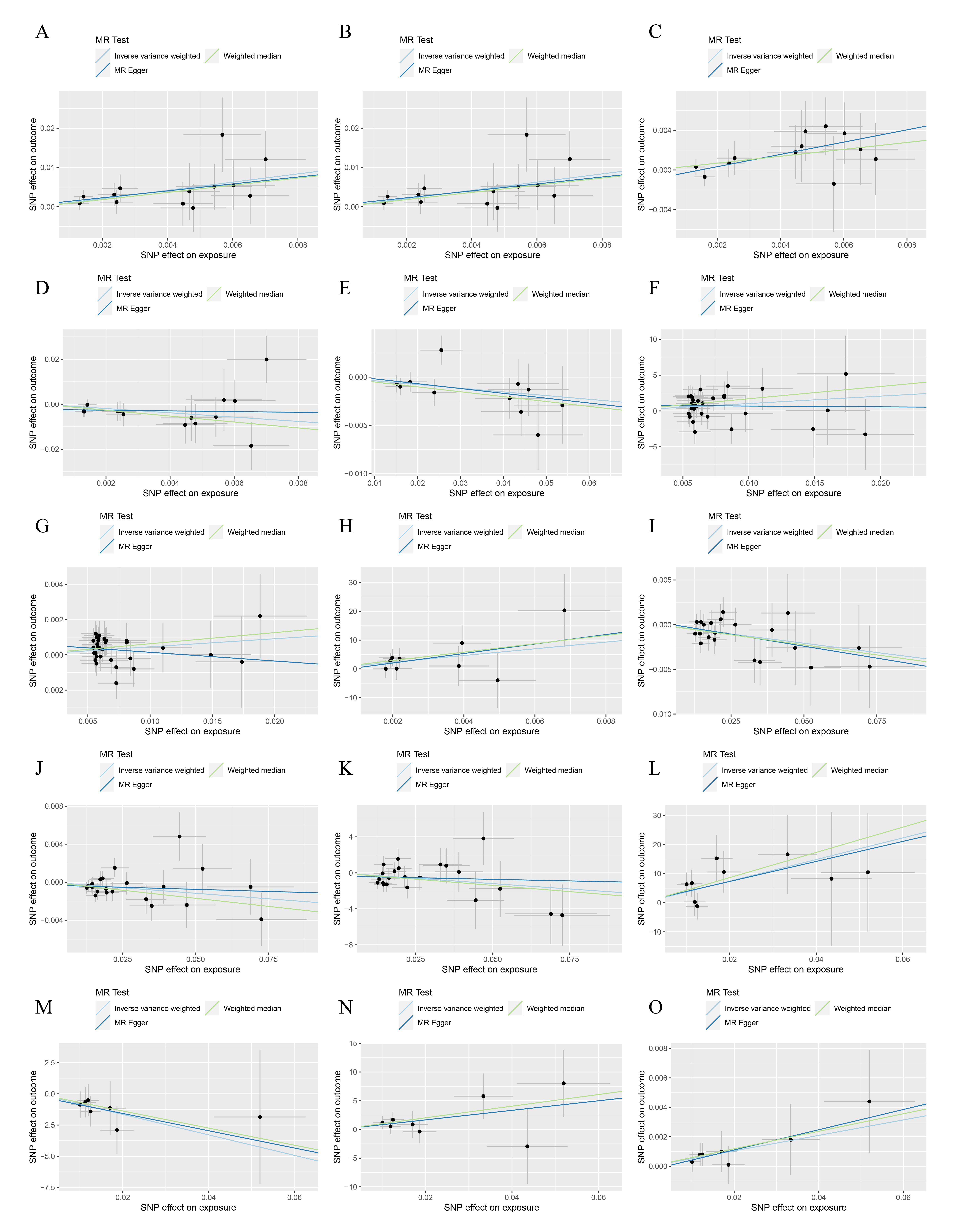
**Figure S1.** Scatter plots of genes predicting nominally significant outcomes of early adversity on cerebral cortex. (A) genetically predicted "Adopted as a child" on TH of caudal anterior cingulate with global weighted; (B) genetically predicted "Adopted as a child" on TH of caudal anterior cingulate without global weighted; (C) genetically predicted "Adopted as a child" on TH of superior temporal with global weighted; (D) genetically predicted "Adopted as a child" on TH of entorhinal with global weighted; (E) genetically predicted "Felt hated by family member as a child" on TH of paracentral with global weighted; (F) genetically predicted "Maternal smoking around birth" on SA of pars triangularis without global weighted; (G) genetically predicted "Maternal smoking around birth" on TH of lateral occipital with global weighted; (H) genetically predicted "Part of a multiple birth" on SA of lateral occipital with global weighted; (I) genetically predicted "Physically abused by family as a child" on TH of banks of the superior temporal sulcus with global weighted; (J) genetically predicted "Physically abused by family as a child" on TH of supramarginal with global weighted; (K) genetically predicted "Physically abused by family as a child" on SA of parahippocampal without global weighted; (L) genetically predicted "Sexually molested as a child" on SA of lateral occipital without global weighted; (M) genetically predicted "Sexually molested as a child" on SA of medial orbitofrontal with global weighted; (N) genetically predicted "Sexually molested as a child" on SA of isthmus cingulate without global weighted; (O) genetically predicted "Sexually molested as a child" on TH of lateral occipital with global weighted.


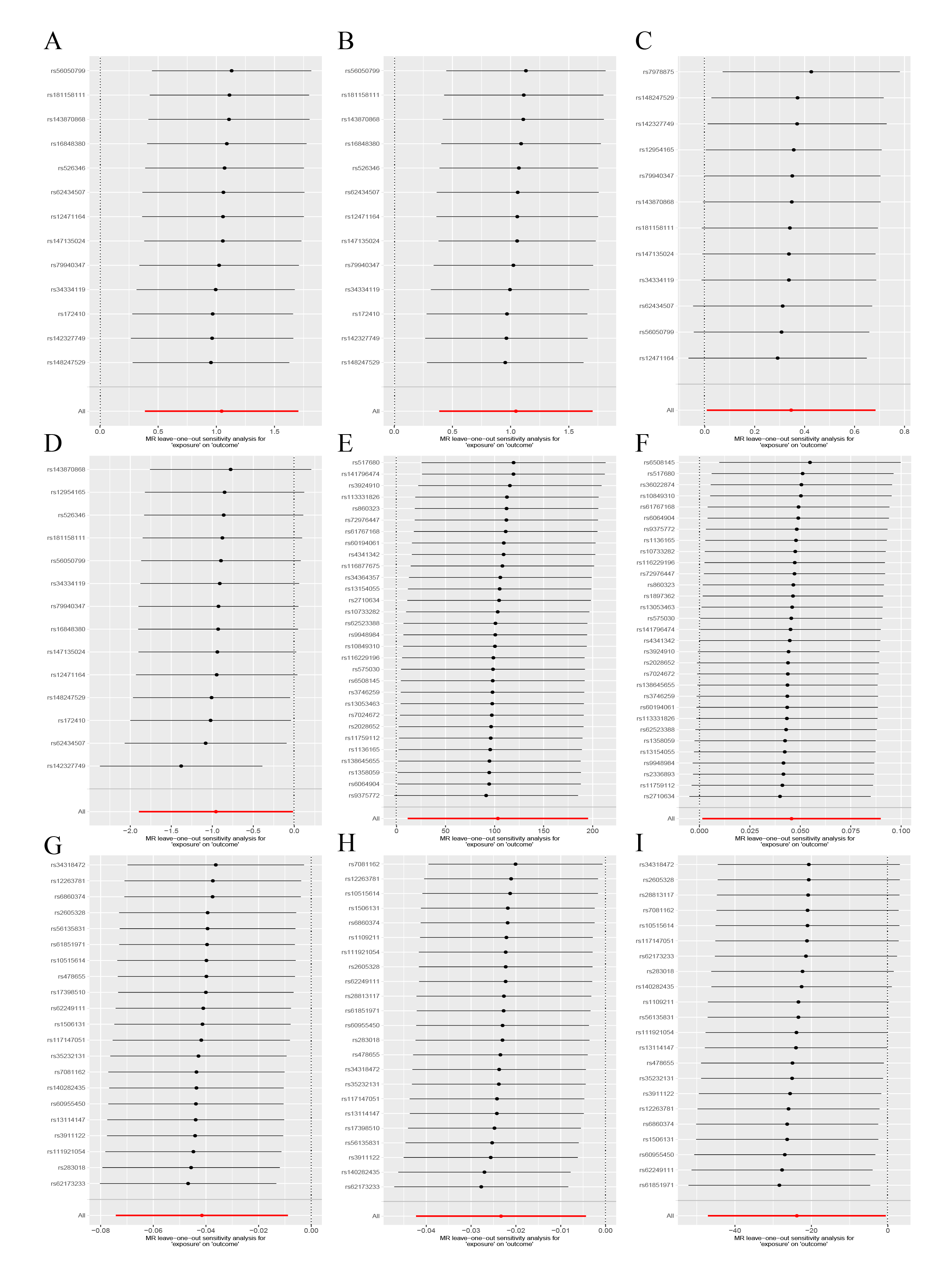


**Figure S2.** Leave-one-out plots of nominally significant outcomes of "Adopted as a child", "Maternal smoking around birth", and "Physically abused by family as a child" on cerebral cortex. (A) genetically predicted "Adopted as a child" on TH of caudal anterior cingulate with global weighted; (B) genetically predicted "Adopted as a child" on TH of caudal anterior cingulate without global weighted; (C) genetically predicted "Adopted as a child" on TH of superior temporal with global weighted; (D) genetically predicted "Adopted as a child" on TH of entorhinal with global weighted; (E) genetically predicted "Maternal smoking around birth" on SA of pars triangularis without global weighted; (F) genetically predicted "Maternal smoking around birth" on TH of lateral occipital with global weighted; (G) genetically predicted "Physically abused by family as a child" on TH of banks of the superior temporal sulcus with global weighted; (H) genetically predicted "Physically abused by family as a child" on TH of supramarginal with global weighted; (I) genetically predicted "Physically abused by family as a child" on SA of parahippocampal without global weighted.


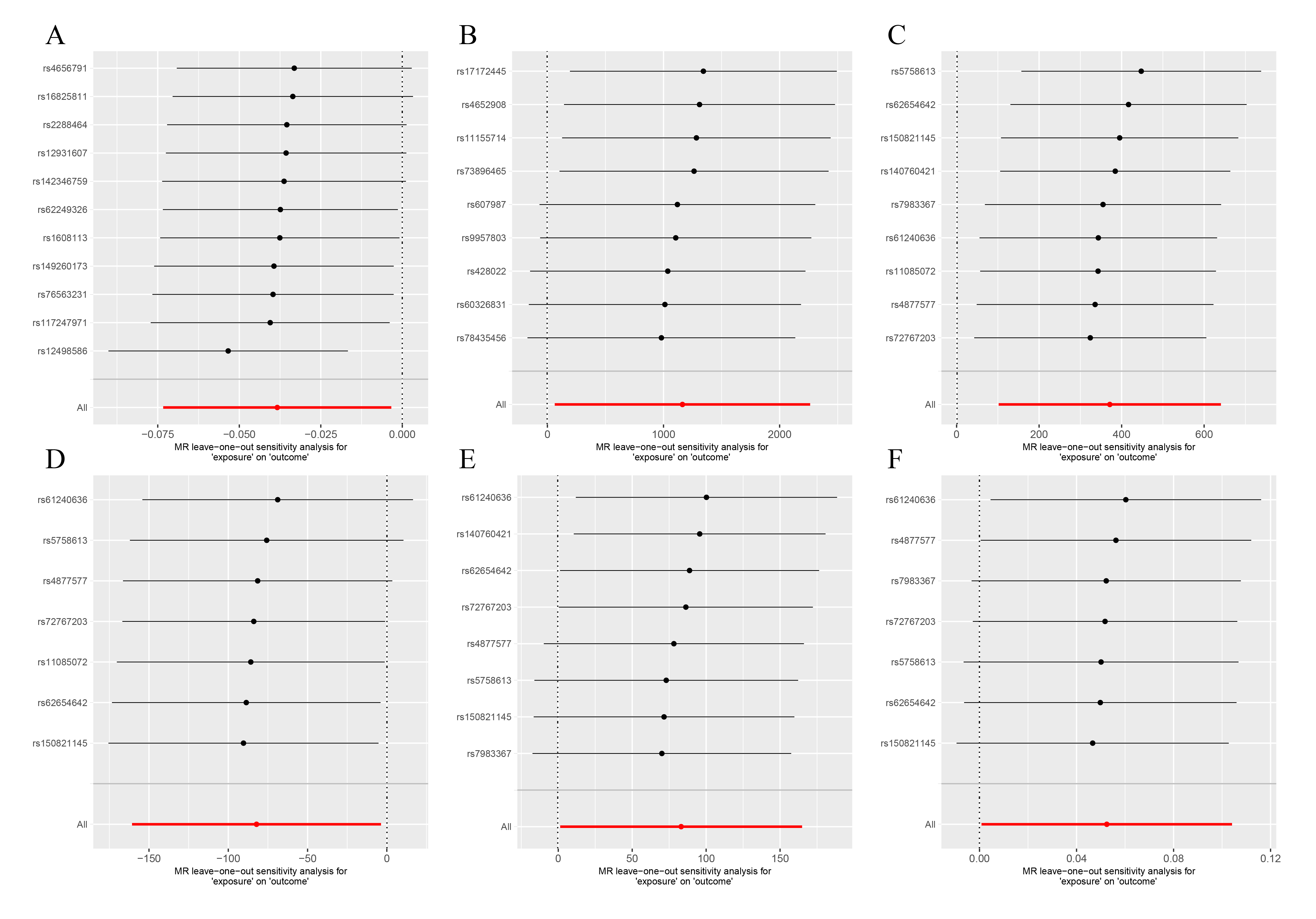


**Figure S3.** Leave-one-out plots of nominally significant outcomes of "Felt hated by family member as a child", "Part of a multiple birth", and "Sexually molested as a child" on cerebral cortex. (A) genetically predicted "Felt hated by family member as a child" on TH of paracentral with global weighted; (B) genetically predicted "Part of a multiple birth" on SA of lateral occipital with global weighted; (C) genetically predicted "Sexually molested as a child" on SA of lateral occipital without global weighted; (D) genetically predicted "Sexually molested as a child" on SA of medial orbitofrontal with global weighted; (E) genetically predicted "Sexually molested as a child" on SA of isthmus cingulate without global weighted; (F) genetically predicted "Sexually molested as a child" on TH of lateral occipital with global weighted.


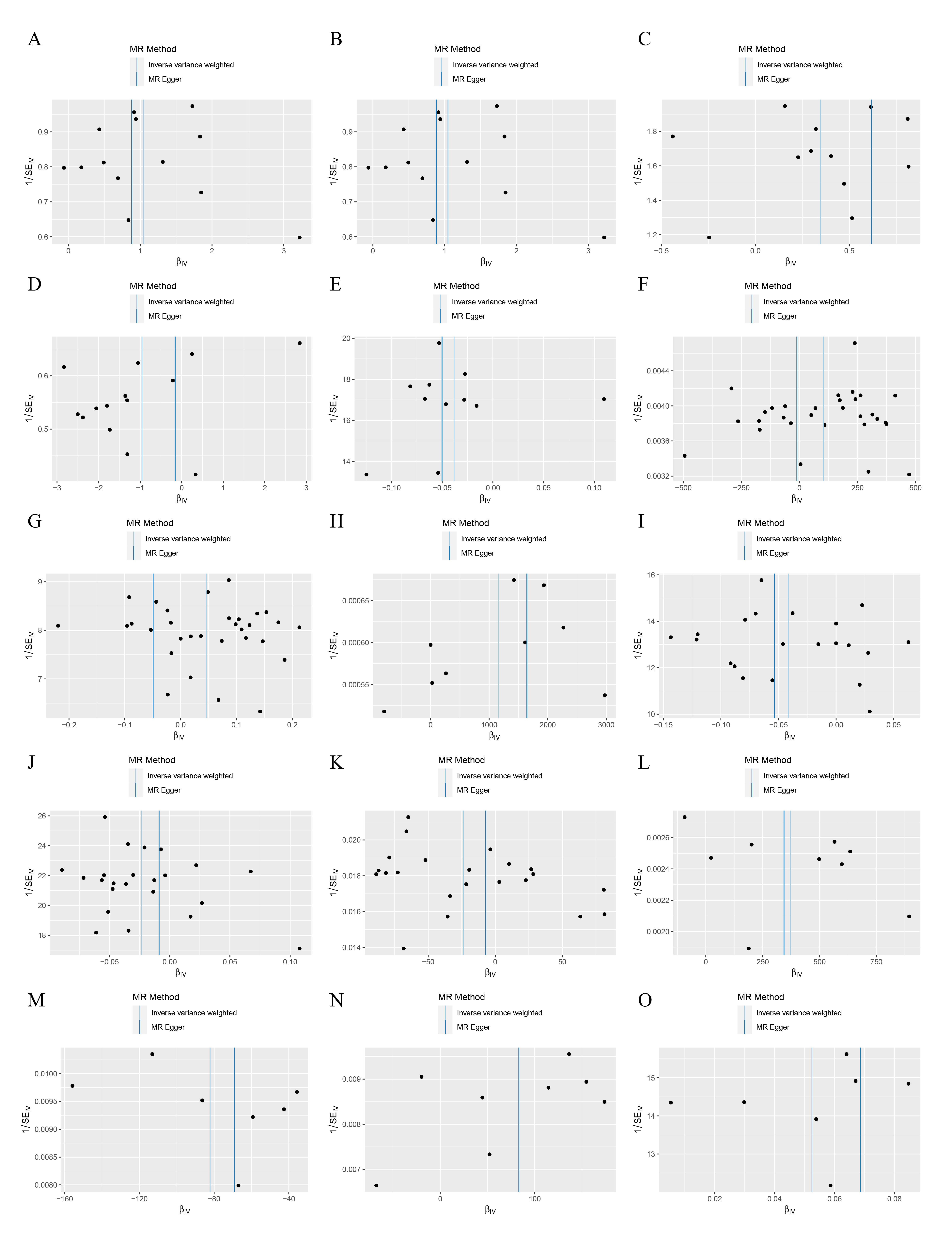


**Figure S4.** Funnel plots of genes predicting nominally significant outcomes of early adversity on cerebral cortex. (A) genetically predicted "Adopted as a child" on TH of caudal anterior cingulate with global weighted; (B) genetically predicted "Adopted as a child" on TH of caudal anterior cingulate without global weighted; (C) genetically predicted "Adopted as a child" on TH of superior temporal with global weighted; (D) genetically predicted "Adopted as a child" on TH of entorhinal with global weighted; (E) genetically predicted "Felt hated by family member as a child" on TH of paracentral with global weighted; (F) genetically predicted "Maternal smoking around birth" on SA of pars triangularis without global weighted; (G) genetically predicted "Maternal smoking around birth" on TH of lateral occipital with global weighted; (H) genetically predicted "Part of a multiple birth" on SA of lateral occipital with global weighted; (I) genetically predicted "Physically abused by family as a child" on TH of banks of the superior temporal sulcus with global weighted; (J) genetically predicted "Physically abused by family as a child" on TH of supramarginal with global weighted; (K) genetically predicted "Physically abused by family as a child" on SA of parahippocampal without global weighted; (L) genetically predicted "Sexually molested as a child" on SA of lateral occipital without global weighted; (M) genetically predicted "Sexually molested as a child" on SA of medial orbitofrontal with global weighted; (N) genetically predicted "Sexually molested as a child" on SA of isthmus cingulate without global weighted; (O) genetically predicted "Sexually molested as a child" on TH of lateral occipital with global weighted.
